# Reply to “Comment on Iavicoli et al. Ethics and Occupational Health in the Contemporary World of Work. *Int. J. Environ. Res. Public Health* 2018, 15, 1713”

**DOI:** 10.3390/ijerph15122708

**Published:** 2018-11-30

**Authors:** Sergio Iavicoli, Antonio Valenti, Diana Gagliardi, Jorma Rantanen

**Affiliations:** 1 Department of Occupational and Environmental Medicine, Epidemiology and Hygiene, Italian Workers’ Compensation Authority (INAIL), Via Fontana Candida 1, Monte Porzio Catone, 00078 Rome, Italy; s.iavicoli@inail.it (S.I.); d.gagliardi@inail.it (D.G.); 2 Department of Public Health/Occupational Health, University of Helsinki, P.O. Box 20 (Tukholmankatu 8 B), FI-00014 Helsinki, Finland; jorma.h.rantanen@gmail.com

The authors would like to extend their thanks for the fruitful comments and suggestions, which are useful for conducting deeper analyses of the ethical concerns related to occupational health.

We were aware of the difficulty of structuring our study due to the limited number of studies found in literature, particularly those focused on the impact of globalisation and the changing world of work on the emerging ethical dilemmas in occupational health, as a testament to the legal awareness about the subject [1].

This represents an important limit, especially given the complexity of the topic discussed in “Ethics and occupational health in the contemporary world of work”, its philosophical, medical, economic, and legal implications, and the interactions between different issues (e.g., (a) the relationship between occupational health and safety on the one hand and labour rights on the other; (b) the impact of the changing world of work, demographic shifts, new technologies, globalization in relation to Occupational Health Professionals’ (OHPs) tasks and professional conduct; and (c) the introduction of new technologies and emerging ethical issues).

We fully agree that several issues could be further addressed and that more discussion is needed on the points of ethics in a globalising working life, as taken up in the commentary [2,3,4]. However, in this type of an article, we face the challenge of limiting the article to the stipulated word limit. This is particularly challenging in the case of such a multidimensional, dynamic issue as ethics in globalisation. For example, including various vulnerable groups of workers would have expanded the text substantially.

Starting from the consideration that there are still few studies about the procedures for addressing ethical issues in occupational health practice, we provided an overview of the main ethical concerns related to the changing world of work, in order to identify “drivers and barriers for correct professional ethics”, also thanks to the ethical analysis of the decision-making process in occupational health practice; this issue has not always been analysed in previous studies.

We analysed the ethical dilemma through an integrated approach, which simultaneously considers the individual, professional, and institutional ethical points of view; for each one, we considered aspects such as the person/body involved, the environment of operation, the philosophical basis, the field of application, value content, the learning arena, and guidance.

To this end, Table 1 is an attempt to visualise the complexity of the issue of occupational health ethics, due, for example, to the number of different actors and stakeholders involved (e.g., when compared to the traditional clinical doctor-patient relationship). It is also intended to demonstrate the differences between personal, professional, and institutional ethics, which are interdependent, may be in harmony, or may fall in conflict with each other. To give an example: We have recently seen an increasing number of cases of ethical misconduct due to the enormous pressure placed on professionals to obtain more funding for research programmes of their institution, and, vice versa, cases in which an individual professional has committed misconducted in the interest of gaining personal credit or money and thereby compromised patient safety and harmed the credibility of their institution. The ICOH amended Code of Ethics proposes a solution for including a paragraph in the working contract of OHPs on entitlement for applying ICOH code in their practice.

The identification of the “next step” for resolving the ethical challenges that OHPs will encounter could represent a starting point for recognising future proposals for ethical solutions, which might also include the engagement of different stakeholders (e.g., reinforcing social dialogue) [5]. Our intention was to stimulate the discussion about emerging ethical issues in occupational health practice in the contemporary world of work, and we are grateful that this seems to have occurred.

We would like to thank the editor for giving us the opportunity to provide a reply to the letter.

## Figures and Tables

**Table 1:** Personal, Professional and Institutional ethics.

**(a). Target**			
	**Personal Ethics**	**Professional Ethics**	**Institutional Ethics**
**Person/body involved**	Individual	Expert	Institution, company, (and their boards and chief executive officers)
**Arena of operation**	Home, private life, community life	Workplace, association, public life	Public environment, business life, community
**(b). Philosophical and cultural bases, values, field of application**			
	**Personal ethics**	**Professional ethics**	**Institutional ethics**
**Philosophical and cultural basis**	Religious ethics, ethnicity, individual humanist ethics, or similar	Deontology	Deontology
**Field of application**	Family, close community, school, workplace	School, university, workplace, professional association, community	Institution, workplace, community, global economy
**Value content**	**Personal values**	**Professional values**	**Five principles of CSR & Global Compact:**
	Honesty	Fairness	**Fair business**
	Trustworthiness	Respect of autonomy	Accountability
	Respect	Beneficence	Transparency
	Responsibility	Non-maleficence	**Human rights (HR)**
	Integrity	Justice	Implementing HR
	Fairness	Competence	Acting against HR abuses
	Compassion, caring	Skill	**Fair employer**
	Courage	Confidentiality	Workers’ rights
			Elimination of inhuman labor
			Anti-discrimination
			**Environment**
			Precautionary principle
			Environmental responsibility and environment-friendly technologies
			**Anti-corruption**
**(c). Guidance and education**			
	**Personal ethics**	**Professional ethics**	**Institutional ethics**
**Learning arena**	Family, school, associations, church	Training institutions, schools, universities, polytechnics, professional stages	University, business school
**Guidance**	Guidance in general upbringing and school or religious education	Professional codes of conduct, Good practice guidelines, Helsinki Declaration, Council for International Organizations of Medical Sciences (CIOMS) guidelines	CSR, United Nations global compact
